# Adjunctive vein of marshall ethanol infusion for pulmonary vein isolation in unilateral left pulmonary artery agenesis: a case report

**DOI:** 10.1093/ehjcr/ytaf682

**Published:** 2026-01-05

**Authors:** Akio Chikata, Takeshi Kato, Kazuo Usuda, Masayuki Takamura

**Affiliations:** Department of Cardiology, Toyama Prefectural Central Hospital, 2-2-78 Nishi-nagae, Toyama 930-8550, Japan; Department of Cardiovascular Medicine, Kanazawa University Graduate School of Medical Science, 13-1 Takara-machi, Kanazawa 920-8641, Japan; Department of Cardiovascular Medicine, Kanazawa University Graduate School of Medical Science, 13-1 Takara-machi, Kanazawa 920-8641, Japan; Department of Cardiology, Toyama Prefectural Central Hospital, 2-2-78 Nishi-nagae, Toyama 930-8550, Japan; Department of Cardiovascular Medicine, Kanazawa University Graduate School of Medical Science, 13-1 Takara-machi, Kanazawa 920-8641, Japan

**Keywords:** Unilateral left pulmonary artery agenesis, Vein of Marshall ethanol infusion, Pulmonary vein isolation, Case report

## Abstract

**Background:**

Isolated unilateral agenesis of the pulmonary artery (UAPA) is an exceptionally rare congenital anomaly. In adults, it may promote structural remodelling and predispose to atrial fibrillation (AF). Pulmonary vein (PV) isolation in UAPA poses unique challenges due to anatomical variations, hypoplastic pulmonary veins (PVs), and altered venous drainage, potentially reducing ablation efficacy.

**Case summary:**

An 80-year-old woman with persistent AF and heart failure, and left UAPA, underwent PV isolation using a size-adjustable cryoballoon (POLARx FIT, Boston Scientific, Marlborough, MA, USA). Computed tomography demonstrated the absence of the left pulmonary artery and systemic collateral supply to the left lung. Left superior PV (LSPV) occlusion with a 31-mm cryoballoon achieved complete contrast seal (nadir −56°C) but failed electrical isolation. A subsequent 28-mm application with similar occlusion and nadir temperature achieved isolation with a prolonged time-to-isolation of 97 s. The hypoplastic left inferior PV (LIPV) was not ablated to avoid stenosis risk. During a second procedure, LSPV reconnection and residual LIPV potentials were detected. Ethanol infusion into the vein of Marshall (VOM) successfully isolated both veins.

**Discussion:**

In UAPA, extensive systemic collateral circulation to the affected lung may cause heat dissipation (‘heat sink effect’), limiting the efficacy of thermal energy delivery. Moreover, hypoplastic PVs carry an increased risk of stenosis after thermal ablation. Additional non-thermal lesion-creation techniques, such as VOM ethanol infusion, may be necessary to achieve durable and safe isolation when conventional thermal methods are insufficient.

Learning points

- In unilateral agenesis of the pulmonary artery (UAPA), anatomical variations, hypoplastic veins, and altered venous drainage may complicate pulmonary vein isolation.
- Extensive systemic collateral circulation can create a ‘heat sink effect’, reducing the efficacy of thermal ablation and potentially requiring alternative energy sources.
- When conventional thermal methods are insufficient, Vein of Marshall ethanol infusion can be an effective adjunctive non-thermal strategy to achieve durable isolation.

## Introduction

Isolated unilateral agenesis of the pulmonary artery (UAPA) is an extremely rare congenital anomaly, often diagnosed in childhood. In case of adult patients, symptoms are associated with lack of blood flow, systemic collaterals together with other cardiovascular anomalies, and can be variable with presenting chest infection, haemoptysis, chest pain, pleural effusion, pulmonary hypertension, and congestive heart failure. The prevalence of UAPA without associated cardiac anomalies ranges from 1 in 200 000 to 1 in 300 000 adults, and UAPA is reported to be approximately twice as common on the right side.^1–3^ UAPA can contribute to structural remodelling and predispose to atrial fibrillation (AF).^4^ Pulmonary vein (PV) isolation in patients with UAPA presents unique challenges, including anatomical variations, hypoplastic PVs, and altered venous drainage patterns that may impede effective ablation.

In this report, we present a case illustrating the challenges encountered during catheter ablation in a patient with AF and left UAPA, where multiple procedures and adjunctive VOM ethanol infusion were crucial for achieving durable and safe PV isolation.

## Summary figure

**Figure:**
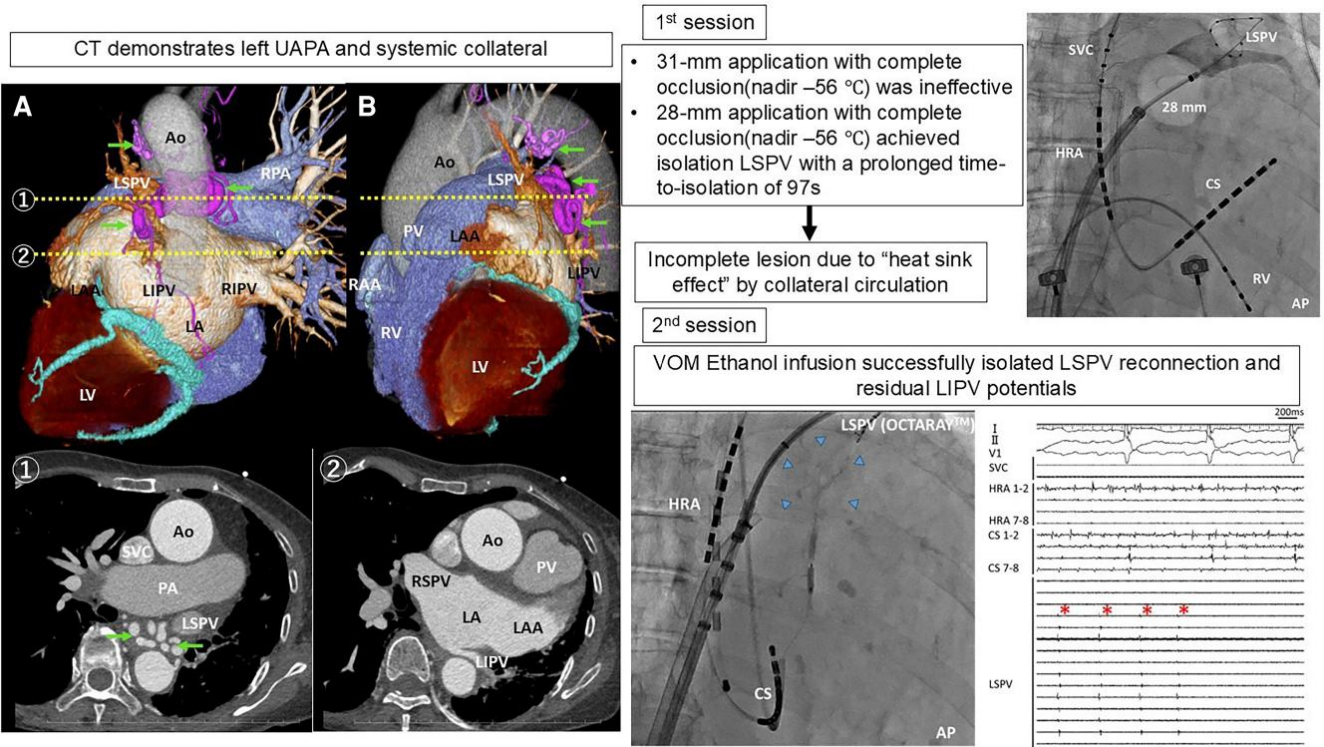


## Case presentation

**Figure 1:**
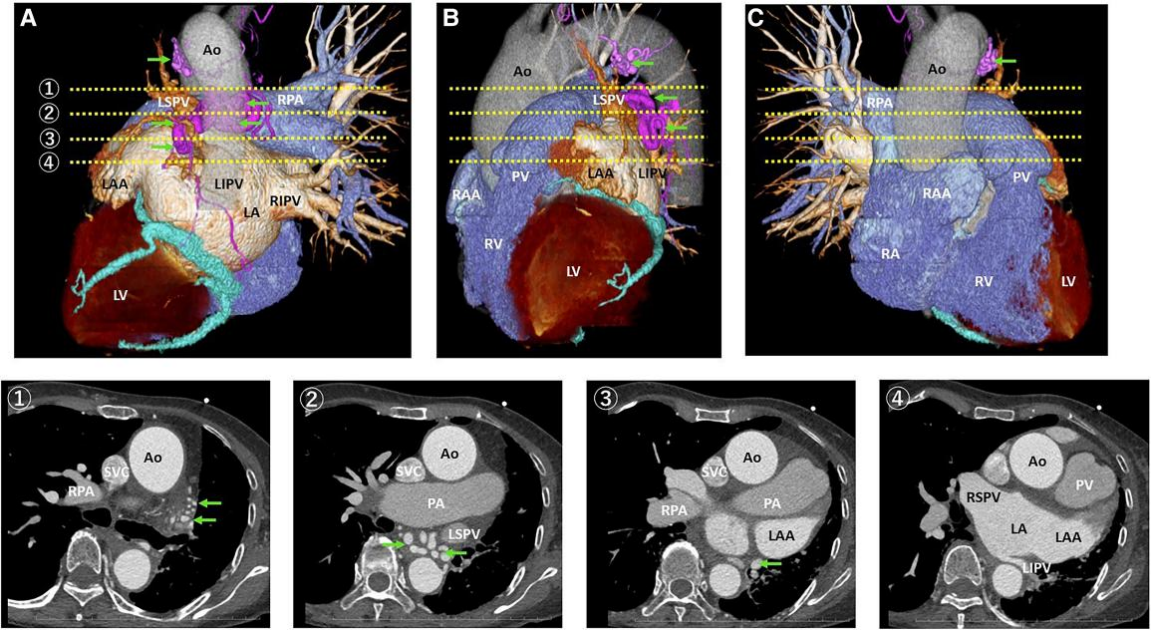
Three-dimensional computed tomography images (*A–C*) and axial slice (1–4). Arrows indicate systemic collateral vessels supplying the left lung. Ao = aorta; LSPV = left superior pulmonary vein; LIPV = left inferior pulmonary vein; RIPV = right inferior pulmonary vein; RPA = right pulmonary artery; PA = pulmonary artery; PV = pulmonary valve; LAA = left atrial appendage; RAA = right atrial appendage; LA = left atrium; RA = right atrium; LV = left ventricle; RV = right ventricle;.

An 80-year-old female with persistent AF and heart failure underwent catheter ablation. She presented exertional dyspnoea corresponding to New York Heart Association (NYHA) class III. Transthoracic echocardiography revealed a left ventricular ejection fraction (LVEF) of 44.6% and diffuse left ventricular wall thickening. Her brain natriuretic peptide (BNP) level was elevated to 892.9 pg/mL, ^99mTc-pyrophosphate scintigraphy demonstrated myocardial tracer uptake, and gastrointestinal endoscopic biopsy demonstrated transthyretin amyloid deposition, confirming transthyretin cardiac amyloidosis (ATTR). Despite optimal guideline-directed medical therapy, including an angiotensin receptor–neprilysin inhibitor (ARNI), mineralocorticoid receptor antagonist (MRA), sodium–glucose cotransporter 2 (SGLT2) inhibitor, and β-blocker, her symptoms persisted. Cognitive function and activities of daily living were well preserved. Considering the persistent symptoms, limited functional capacity, and poor long-term efficacy of pharmacological rhythm control or electrical cardioversion, we elected to perform catheter ablation to restore sinus rhythm and improve heart failure status.　Preprocedural computed tomography imaging demonstrates left UAPA. The left pulmonary artery is absent, and systemic collateral vessels supplying the left lung are evident (arrows), originating primarily from the descending aorta. The left inferior PV (LIPV) appeared markedly small and hypoplastic compared with the other pulmonary veins (*Figure 1*).

**Figure 2:**
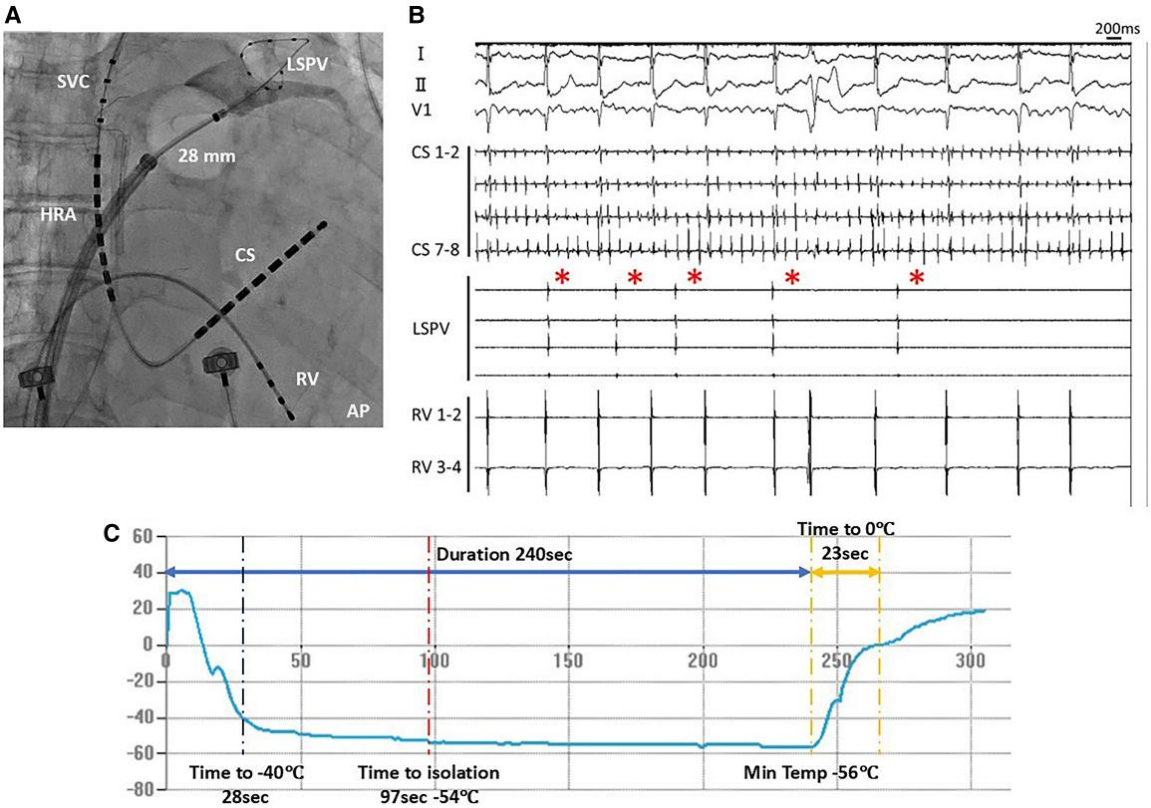
Fluoroscopic image (*A*), intracardiac electrocardiogram (*B*), and balloon temperature change during application for LSPV with POLARx FIT 28 mm balloon (*C*). LSPV = left superior pulmonary vein; RV = right ventricle; HRA = right atrium; CS = coronary sinus.

PV isolation using size-adjustable cryoballoon (The POLARx FIT, Boston Scientific, Marlborough, MA, USA) was performed under conscious sedation. Left superior PV (LSPV) occlusion using a 31-mm cryoballoon achieved a good contrast seal and a nadir temperature of −56°C, but electrical isolation of the LSPV was not achieved. A subsequent application using a 28-mm balloon, with similarly good occlusion (*Figure 2A*) and the same nadir temperature, resulted in initial isolation with a prolonged time-to-isolation (TTI) of 97 s (*Figure 2B* and *C*). The LIPV was hypoplastic, and cryoablation was avoided during the first session due to concerns regarding stenosis or deformation associated with thermal energy delivery.

**Figure 3:**
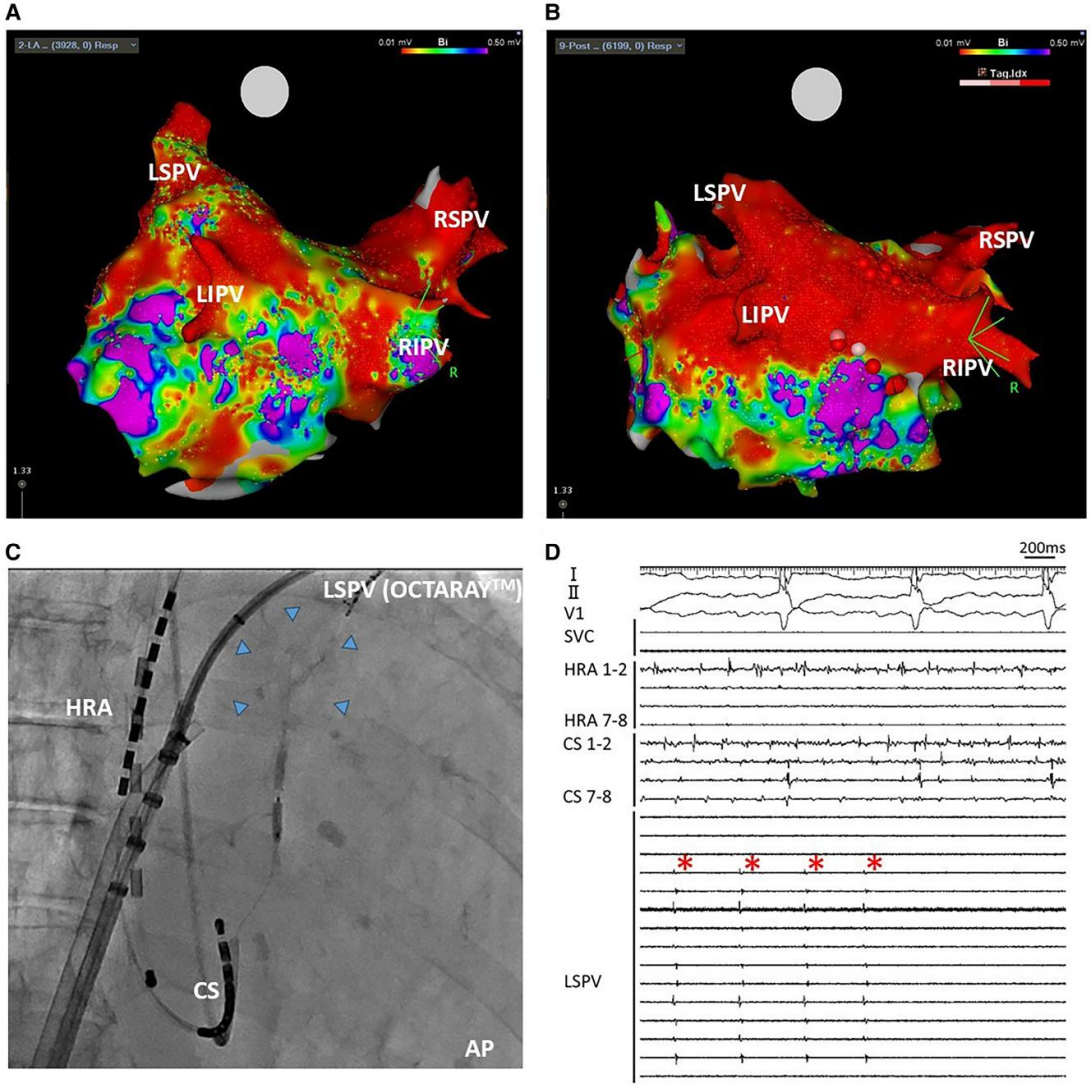
Electroanatomical mapping of pre (*A*) and post (*B*) procedure of second ablation session. Fluoroscopic image (*C*) and intracardiac electrocardiogram (*D*) during ethanol infusion into the VOM. VOM ethanol infusion was performed as follows: using a right internal jugular vein approach, the CS was cannulated with a JR 4.0 catheter, and a Runthrough guidewire (Terumo, Tokyo, Japan) was advanced into the VOM. Subsequently, an angioplasty balloon (Emerge, length 8 mm, nominal diameter 2.0 mm; Boston Scientific, Marlborough, MA) was positioned in the proximal VOM, and a total of 10 mL of 96% ethanol was slowly infused through the balloon to achieve ethanol ablation. Superior vena cava isolation was also performed, and the total procedural time was 156 min. LSPV = left superior pulmonary vein; HRA = right atrium; CS = coronary sinus; VOM = vein of Marshall.

She underwent a second ablation session 6 months later due to a recurrence of AF. During the second ablation session, reconnection of the LSPV, RIPV, and residual electrical activity in the hypoplastic LIPV were observed (*Figure 3A*). Because an epicardial connection was suspected and endocardial ablation using thermal energy alone was considered insufficient, ethanol infusion into the vein of Marshall (VOM) was selected to achieve effective lesion formation while avoiding thermal deformation of the hypoplastic LIPV. LSPV signals were monitored during ethanol infusion into the VOM and eliminated in real time (*Figures 3C* and *D*). LIPV activity was evaluated after infusion completion, confirming no residual potentials. These findings suggest that reconnection of the LSPV and persistent LIPV activity were mediated by conduction potentially protected by the heat sink effect of systemic collateral flow associated with UAPA. VOM ethanol infusion effectively targeted these regions. A voltage map acquired after completion of the VOM infusion, re-isolation of the RIPV, and creation of both roof and bottom lines demonstrated successful box isolation (*Figure 3B*). Unfortunately, AF recurred. After the second recurrence of AF, amiodarone was introduced, and AF transitioned to atrial tachycardia. She underwent a third session 8 months later, but at that time, no reconnection was observed in either LSPV or LIPV. During the third session, both mitral atrial flutter and atrial tachycardia circulating around the anterior wall of the left atrium were confirmed. Restoration of sinus rhythm was achieved by applying radiofrequency energy to the mitral isthmus and the anterior wall of the left atrium. Since then, sinus rhythm has been maintained, and heart failure management has remained stable.

## Discussion

In this report, we present a case with left UAPA who underwent PV isolation for AF. PV isolation using CB balloon could not create durable lesion in the left PV, and adjunctive VOM ethanol infusion were crucial for achieving durable PV isolation.

Two types of presentations are described in patients with UAPA. The first presentation is the one seen in infants, where they usually present with congestive cardiac failure and pulmonary hypertension. The other is in older patients, who are often asymptomatic; they present with exercise intolerance, haemoptysis, and recurrent pulmonary infections or are incidentally detected during chest radiography.^3^ Although extensive systemic collateral circulation to the affected lung may cause haemoptysis but may promote heat dissipation from the epicardium in case of catheter ablation from the endocardial side using thermal energy—a phenomenon known as the ‘heat sink effect’—and impair effective thermal energy-based energy delivery.^5^ During the first ablation session, PV isolation using size-adjustable cryoballoon (POLARx FIT) was performed and PV isolation was unsuccessful at POLARx FIT 31 mm but temporarily successful at POLARx FIT 28 mm despite achieving good occlusion in both balloons. Among commercially available balloons, the POLARx FIT of 28 mm has been reported to achieve the greatest tissue temperature drop;^6^ the fact that the transient effect was only observed at POLARx FIT 28 mm suggests that thermal energy delivery to deeper layers was impeded by collateral circulation.

Thermal energy also has potential risk of PV stenosis and deformation. Therefore, we considered that repeated ablation using thermal energy for LSPV and thermal application for the hypoplastic LIPV were undesirable. VOM ethanol infusion not only creates the endocardial and epicardial lesions around the vessel but also contribute achievement of left PV isolation.^7–9^ Thus, VOM ethanol infusion was considered as a possible adjunctive therapy if complete isolation could not be achieved with cryoballoon ablation and recurrence of AF. In the present case, adjunctive VOM ethanol infusion contributed to avoid potential risk of PV stenosis and deformation caused by thermal energy and incomplete lesion formation of the epicardial side by heat sink effect of systemic collateral circulation.

Recently, PFA has emerged as a novel non-thermal energy source for AF ablation. Unlike thermal modalities, PFA selectively targets myocardial tissue through electroporation, thereby minimizing collateral injury and reducing the risk of PV narrowing or stenosis.^10^ These characteristics suggest that PFA may be advantageous in patients with UAPA, where extensive systemic collateral circulation can cause the ‘heat sink effect’ and impair the efficacy of thermal energy delivery.

However, several experimental and clinical studies have also reported the occurrence of coronary vasospasm and, in some instances, chronic vascular narrowing when PFA is applied near arterial segments.^11–14^ In the present case, the systemic collateral vessels supplying the left lung were derived from arterial sources and therefore likely contained vascular smooth-muscle components. This raised theoretical concerns regarding vasospasm-induced haemodynamic deterioration, worsening of ventilation–perfusion mismatch, or late vascular remodelling leading to haemoptysis. Therefore, although PFA may offer potential benefits in such complex anatomy, its safety in the presence of arterial collateral circulation should be carefully evaluated before clinical application.

## Conclusion

When PV isolation is required for left UAPA patients, adjunctive strategies especially for epicardial lesion creation and avoidance of thermal deformation may be necessary to create safety and durable lesion. VOM ethanol infusion was deemed a safe and effective treatment option.

## Data Availability

Data will be made available from the authors on reasonable request. **IRB:** Toyama Prefectural Central Hospital Ethics Committee (no. 67–77)

## Lead author biography

Dr Akio Chikata obtained his MD and PhD degrees from the University of Kanazawa in Kanazawa, Japan. He has been working as a cardiologist specializing in electrophysiology at Toyama Prefectural Central Hospital since 2016.

**Consent:** The authors confirm that written consent for submission and publication of this case report associated text has been obtained from the patient in line with COPE guidelines.
